# Nuchal translucency: an ultrasound marker for fetal chromosomal abnormalities

**DOI:** 10.1590/S1516-31802001000100006

**Published:** 2001-01-02

**Authors:** Gregório Lorenzo Acácio, Ricardo Barini, Walter Pinto, Renato Luís Silveira Ximenes, Heverton Pettersen, Marcos Faria

**Keywords:** Aneuploidy, Ultrasound, Chromosomes, Fetus, Prenatal Diagnosis, Aneuplodia, Ultra-sonografia, Cromossomos, Feto, Diagnóstico pré-natal

## Abstract

**CONTEXT:**

The literature shows an association between several ultrasound markers and chromosome abnormality. Among these, measurement of nuchal translucency has been indicated as a screening method for aneuploidy. The trisomy of chromosome 21 has been most evaluated.

**OBJECTIVE:**

To define the best fixed cutoff point for nuchal translucency, with the assistance of the ROC curve, and its accuracy in screening all fetal aneuploidy and trisomy 21 in a South American population.

**TYPE OF STUDY:**

Validation of a diagnostic test.

**SETTING:**

This study was carried out at the State University of Campinas, Campinas, Brazil.

**PARTICIPANTS:**

230 patients examined by ultrasound at two tertiary-level private centers, at 10 to 14 weeks of gestation.

**DIAGNOSTIC TEST:**

The participants consisted of all those patients who had undergone ultrasound imaging at 10 to 14 weeks of gestation to measure nuchal translucency and who had had the fetal or neonatal karyotype identified.

**MAIN MEASUREMENTS:**

Maternal age, gestational age, nuchal translucency measurement, fetal or neonatal karyotype.

**RESULTS:**

Prevalence of chromosomal defects – 10%; mean age – 35.8 years; mean gestational age – 12 weeks and 2 days; nuchal translucency (NT) thickness – 2.18 mm. The best balance between sensitivity and specificity were values that were equal to or higher than 2.5 mm for overall chromosomal abnormalities as well as for the isolated trisomy 21. The sensitivity for overall chromosomal abnormalities and trisomy 21 were 69.5% and 75%, respectively, and the positive likelihood ratios were 5.5 and 5.0, respectively.

**CONCLUSION:**

The measurement of nuchal translucency was found to be fairly accurate as an ultrasound marker for fetal abnormalities and measurements equal to or higher than 2.5 mm were the best fixed cutoff points.

## RESUMO

**CONTEXTO:**

A literatura mostra uma associação entre diversos marcadores ultra-sonográficos e riscos de cromossomopatias. Dentre os marcadores ultra-sonográficos, a medida da translucência nucal têm sido apontada como um método de rastreamento de aneuploidias, sendo a trissomia do cromossomo 21 a mais investigada.

**OBJETIVO:**

Definir, com auxilio da curva ROC, o melhor ponto de corte fixo da translucência nucal e sua acurácia no rastreamento das aneuploidias fetais como um todo, e para a trissomia do cromossomo 21 numa população sul americana.

**TIPO DE ESTUDO:**

Validação de teste diagnóstico.

**LOCAL:**

O estudo foi realizado na Universidade Estadual de Campinas, Campinas, Brasil.

**PARTICIPANTES:**

230 pacientes que realizaram ultra-sonografia em dois centros privados de nível terciário, entre 10 e 14 semanas de gestação.

**PROCEDIMENTOS:**

Foram incluídas as pacientes que realizaram ultra-sonografia, entre 10 e 14 semanas de gestação, onde se mediu a translucência nucal, e a pesquisa do cariótipo fetal ou do recém-nascido.

**VARIÁVEIS ESTUDADAS:**

Idade materna, idade gestacional, medida da translucência nucal e resultado do cariótipo fetal ou do recém-nascido.

**RESULTADOS:**

A prevalência de cromossomopatias foi de 10%. A idade média das pacientes foi de 35,8 anos. A idade gestacional média foi de 12 semanas e dois dias. A medida da translucência nucal (TN) apresentou uma média de 2,18 mm. O ponto de corte fixo, com melhor equilíbrio entre sensibilidade e especificidade, foram os valores iguais ou maiores que 2,5 mm para todas as cromossomopatias e, também, para a trissomia do cromossomo 21 isoladamente. A sensibilidade do teste foi de 69,5% e 75% para todas as cromossomopatias e para a trissomia do cromossomo 21 respectivamente, com uma razão de verossimilhança positiva de 5,5 e 5,0 para todas as cromossomopatias e para a trissomia do cromossomo 21 respectivamente.

**CONCLUSÕES:**

A medida translucência nucal mostrou boa acurácia como marcador ultra-sonográfico de cromossomopatias fetais, sendo o valor de 2,5 mm o melhor ponto de corte fixo.

## INTRODUCTION

Szabó & Gellen^1^ were the first to report a relationship between accumulated fetal nuchal fluid and fetal abnormalities. They observed 105 normal karyotype fetuses and found more than 3 mm of nuchal fluid accumulated in 7 fetuses with trisomy 21 and in one normal karyotype fetus.

In 1992, Nicolaides et al.^2^ introduced the new term “nuchal translucency (NT)” which was defined as the thickness of the translucent space between the skin and the soft tissue over-lying the fetus cervical spine, measured in millimeters and tenths of a millimeter via ultrasound. This study was performed in a high-risk population sample, at 10 weeks to 14 weeks pregnant. The NT measures considered abnormal were 3 mm and above and with 64% sensitivity for trisomy 21.

The etiology for this nuchal fluid accumulation has still not been defined and various theories offer an explanation. The most cited are: deficient and transitory lymphatic drainage of the cervical region due to disorders in the lymphatic connections,^3,4^ excessive perfusion of the protective mechanism of the central nervous system as a result of the rapid growth of the initial placenta which consequently increases the circulatory volume^5^ and cardiac alterations - mainly the narrowing of the aortic isthmus and consequently increasing the vascular flow of the fetal cervical region.^6,7^

Many authors^8-17^ have published studies showing that an increase in the nuchal thickness measured in the first and at the beginning of the second trimester of pregnancy is associated with greater prevalence of fetal aneuploidy. The sensitivity of these publications with different cutoff points varies from 20% to 93.5%.

Some publications consider NT ^3^ 2.5 mm^18,19^ as positive screening values, whereas the great majority use a fixed value of -NT ^3^ 3 mm.^2,10,15,20,21,22^

The Harris Birthright Research Centre for Fetal Medicine has coordinated the largest study to assess NT accuracy. It was conducted at 22 ultrasound centers in England on 96,127 women who were 10 weeks to 14 weeks pregnant. The risk for trisomy 21 was calculated by multiplying the NT probability ratio by the prevalence of this trisomy at different maternal and gestational ages. The test was positive for 5% of the population which included 77% of the trisomy 21 cases.^23^ This study considered the patient's age as well as the gestational age and used a software program to calculate the risk for trisomy 21.

The aim of this study was to define the best fixed cutoff point for nuchal translucency, with the assistance of the receiver operator characteristic curve (ROC curve),^24^ and the accuracy of this cutoff for all fetal aneuploidy screening and for trisomy of chromosome 21 in a South American population.

## METHODS

The study was submitted for assessment to the Research Ethics Committee of the State University of Campinas (UNICAMP) and was approved.

This was a diagnostic validation study. The sample size was estimated using Schäfer's^24^ method – a specific method for validation studies that uses the ROC curve assuming a 70% sensitivity and 90% specificity.^25^ A minimum of 217 participants were necessary.

The study included patients who had undergone ultrasound imaging at tertiary level private centers and who, according to the crown-rump length (CRL),^26^ were at the stage of between 10 and 14 weeks gestation, with a single gestation and live fetus. Nuchal translucency was measured in all the cases and later, fetal karyotyping was carried out for indications that excluded abnormal NT.

**Figure 1:**
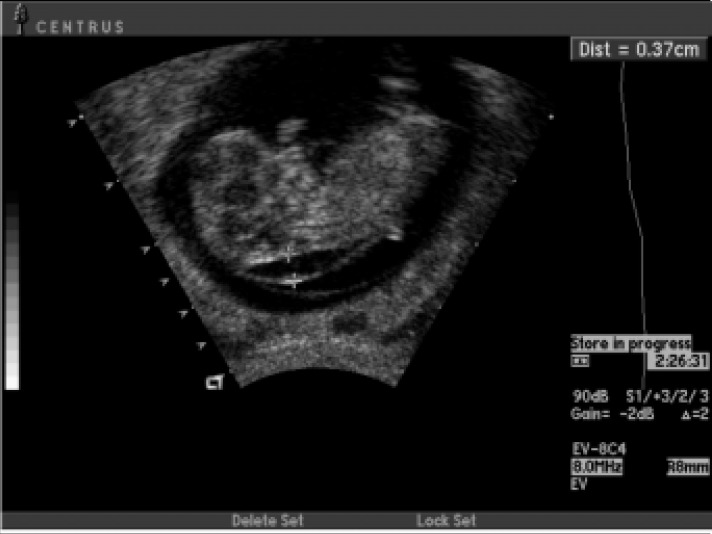
Ultrasound image of the caliper method used for measuring nuchal translucency.

Nuchal translucency was defined as the thickness of the translucent space between the skin and the soft tissues overlying the fetus cervical spine, measured in millimeters and tenths of a millimeter by ultrasound and following the criteria set by the Fetal Medicine Foundation^27^ (Figure 1).

Five physicians certified by the London or Brazilian Fetal Medicine Foundation measured the CRL and NT using the Sequoia®, Aspen 128 X P 10 - Acuson® and Toshiba® SH 140 equipment. The ultrasound examination was transvaginal or abdominal.

Chorionic villus biopsy, amniocentesis, blood or placenta provided fetal cells for fetal karyotyping.

A univariate descriptive analysis was conducted and the sensitivity, specificity and the nuchal translucency ratio were calculated for normal or altered karyotype (the gold standard).

The ROC curve for the nuchal translucency values was drawn in order obtain the best cutoff point for this measure. Logistic regression analysis was used to find out whether the translucency value was a predictor for fetal abnormalities. All the data used in this study were obtained from the files of patients who had previously undergone nuchal translucency measurement and fetal karyotyping.

## RESULTS

The study included 230 patients. There was a 10% prevalence of fetal abnormalities (Table 1). The patients’ ages ranged from 21 years to 45 years – the mean age was 35.8 years. The minimum crown-rump length of the fetus (CRL) was 39 mm (which corresponds to a 10-week gestational age) and its maximum value was 86 mm (14 weeks gestational age). The mean CRL was 59.7 mm (12 weeks and 2 days gestational age) (Table 2). The mean value of nuchal translucency (NT) was 2.18 mm – the minimum value was 0.9 mm and the maximum value was 14 mm (Table 3).

**Table 1:** Distribution according to karyotype fetus

	Normal		Affected	
	*N*	%	*N*	%
**Total**	**207**	**90**	**23**	**10**

**Table 2:** The Crown-Rump Length (CRL)

	N	Average (mm)	*CI (95%)	Minimum (mm)	Maximum (mm)
**Total**	**230**	**59.70**	**58.30 to 61.08**	**39.0**	**86.0**

* *confidence interval.*

**Table 3:** Nuchal translucency values

	N	Average (mm)	*CI (95%)	Minimum (mm)	Maximum (mm)
**Total**	**230**	**2.18**	**1.96 to 2.39**	**0.90**	**14.00**

* *confidence interval.*

A higher frequency of normal karyotype fetuses was observed when the NT values were low and when the NT values were higher; the frequency of aneuploidy was also higher (P < 0.01) (Table 4). The most frequent chromosomal abnormality was trisomy 21 followed by trisomy 18 (Table 5).

**Table 4:** Fetal karyotype according to nuchal translucency

Translucency (mm)	Normal		Affected	
	n	%	n	%
< 1	3	1.5	0	-
1.0ú¾1.5	50	24.1	1	4.3
1.5ú¾2	94	45.5	5	21.8
2.0ú¾2.5	34	16.5	1	4.3
2.5ú¾3.0	14	6.7	2	8.7
3.0ú¾4.0	9	4.3	2	8.7
4.0ú¾5.0	1	0.5	2	8.7
5.0ú¾10.0	2	0.9	7	30.5
10 or more	0	-	3	13.0
**Total**	**207**	**100.0**	**23**	**100.0**

*Cochran-Armitage tendency test: P < 0.01.*

**Table 5:** Maternal age, gestational age (GI), nuchal translucency (NT) and fetal karyotype with aneuploidies

Case number	Maternal age (years)	GA (weeks)	NT (mm)	Karyotype
1	26	11 weeks	1.0	47,XY,+21
2	26	11 weeks and 4 days	1.7	69,XXY
3	34	14 weeks	1.8	92,XXYY
4	41	13 weeks and 4 days	1.8	46,XX /45,X0
5	36	12 weeks and 5 days	1.8	47,XY,+21
6	37	12 weeks and 5 days	1.8	47,XY,+18
7	42	12 weeks	2.0	47,XY,+21
8	34	12 weeks and 4 days	2.5	47,XY,+21
9	41	11 weeks and 6 days	2.5	47,XX,+18
10	42	12 weeks and 4 days	3.0	47,XY,+18
11	38	11 weeks and 3 days	3.8	47,XX,+21
12	41	12 weeks	4.5	47,XX,+7
13	41	11 weeks and 6 days	4.8	47,XY,+21
14	38	13 weeks and 5 days	5.1	47,XY,+21
15	30	11 weeks and 2 days	5.3	47,XY,+21
16	38	11 weeks and 1 day	6.9	45,X0
17	42	12 weeks	7.2	47,XX+21
18	25	13 weeks and 5 days	7.7	47,XX,+21
19	38	12 weeks and 3 days	8.8	47,XX,+21
20	37	11 weeks and 2 days	9.4	47,XX+18
21	41	13 weeks and 5 days	10.0	47,XY,+21
22	26	13 weeks and 6 days	12.0	45,X0
23	43	13 weeks and 5 days	14.0	47,XY,+18

**Figure 2:**
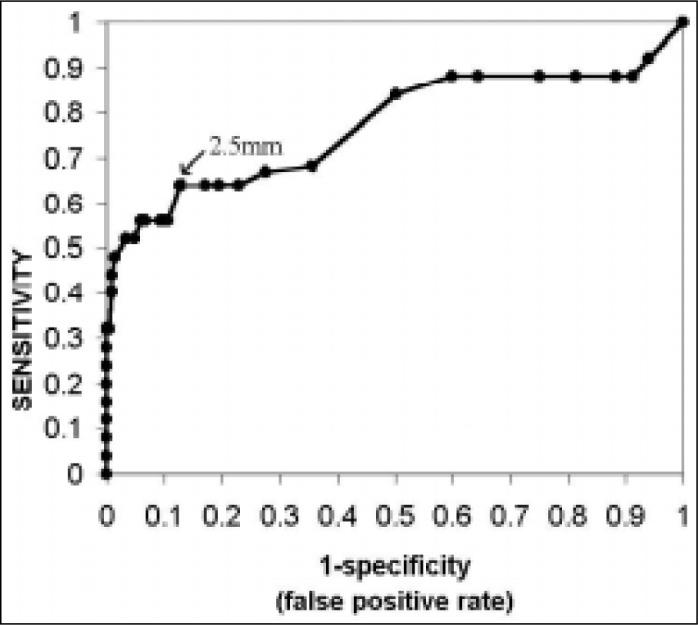
Balance between NT sensitivity and specificity to screen all chromosomal abnormalities at different cutoff points of Receiver Operator Characteristic Curve (ROC).

**Figure 3:**
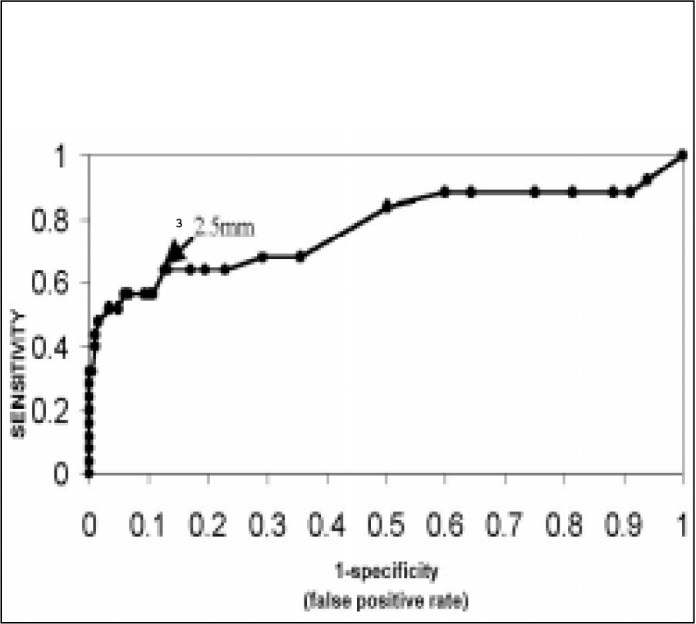
Balance between NT sensitivity and specificity for trisomy 21 at different cutoff points of Receiver Operator Characteristic Curve (ROC).

The ROC curve (Figure 2) was drawn based on the sensitivity and specificity values and identified NT ^3^ 2.5 mm as a good balance between sensitivity and specificity for screening aneuploidy. The measurement of NT ^3^ 2.5 mm helped to correctly classify 16 fetuses out of 23 fetuses with chromosomal alterations and 181 fetus out of 207 normal karyotype fetuses (Table 6). The ROC curve also demonstrates that an NT measurement ^3^ 2.5 mm gives a good balance between sensitivity and specificity for screening trisomy 21 (Figure 3). Using the cut-off point of NT ^3^ 2.5 mm (Table 7), 9 out of 12 fetuses with trisomy 21 (75% sensitivity) and 185 out of 218 normal fetuses (84.9% specificity, 5.0 likelihood ratio) were correctly classified.

**Table 6:** Altered fetal karyotype and normal karyotype - proportion at the chosen cutoff point

	Altered	Normal
TN ^3^ 2.5	16	26
TN < 2.5	7	181
**Total**	**23**	**207**

*Sensitivity = 69.5%; Specificity = 87.4%; Positive likelihood ratio = 5.5.*

**Table 7:** Fetuses with trisomy 21 and those without, according to the chosen cutoff point

	Present (n)	Absent (n)
TN ^3^ 2.5	9	33
TN < 2.5	3	185
**Total**	**12**	**218**

*Sensitivity = 75.0 %; Specificity = 84.9 %; Positive likelihood ratio 5.0.*

## DISCUSSION

This study proved that nuchal translucency at 10 to 14 weeks gestation was useful for screening general chromosomal abnormalities as well for specific trisomy 21 screening.

The mean age of the women in this study was 35.8 years. This age group showed a high risk for aneuploidy and, as such, this fact may have positively influenced the accuracy of the test. There can be two explanations for the 10% aneuploidy prevalence – the women's age and a concentration of high risk cases at the two clinics, which were Fetal Medicine referral centers.

The tendency tests used showed that the NT measurement for normal fetuses was lower than that for fetuses that had chromosomal abnormalities. This was in keeping with the data in the literature^10,17,19,28-32^ and reinforced the use of the NT measure for screening aneuploidy.

The ROC curve helped define the best cutoff point for the NT measurement at ^3^ 2.5 mm^18,19^ for all aneuploidy. Some other studies have also obtained the same NT value, but a greater number of research studies have fixed the value as NT ^3^ 3 mm.^2,10,15,20-22^

When the NT measurement ^3^ 2.5 mm, all the aneuploidy showed a 69.5% sensitivity, 87.4% specificity and 5.5 likelihood ratio. These results coincided with the 65% sensitivity obtained by Hafner et al.^19b^ in a 0.4% aneuploidy population and also with the results of Pandya et al.^18^ - 75% sensitivity and 0.2% aneuploidy.

The most frequent chromosomal alteration was trisomy 21 (12 cases) followed by trisomy 18 (5 cases), and in 3 cases the chromo-some X monosomy, one of which was a case of mosaicism. Trisomy 21 is also the most frequent occurrence in the literature. In neonates, its frequency of identification is tenfold when compared to trisomy 18,^33^ and as in this study, three times more frequent in 9 to 14 week gestations.^34^ In the literature, the frequency of the X monosomy is 1.5% of all recognized gestations, although only 1% of these survive beyond the 28^th^ gestational week. The proportions of trisomy 21, trisomy 18 and X monosomy were similar to those found by Snijders et al.^23^ in 1998.

The largest collaborative study published to date on nuchal translucency consisted of 96,127 patients, used sequential risk, and its accuracy for trisomy 21 is better than that found in this study (82.2%). However, software is needed to classify the positively and negatively screened cases. Probably the reason for the high accuracy of this large collaborative study by Snijders is that it took into account the risk related to gestational age, maternal age and the nuchal translucency measure when calculating sequential risk.

The criteria for sample selection had the aim of reducing the verification bias^36^ by including in the study only those cases where the indication had not been an increased NT but the karyotype as a definite diagnosis. However, the number of cases where the indication for fetal karyotype was unknown was high. Maybe the fetal karyotype was studied in these cases because the NT measure was high.

Studies published with large samples have used NT as an indication of fetal karyotype and karyotype for high risk patients only (advanced maternal age, previous malformations), whereas the assessment of the perinatal phenotype has been reserved for only low risk cases and low NT.^2,10,15,18-23^

The abortion rate is higher when the fetal NT is high and chromosomal abnormalities are present.^37^ Therefore, the neonate pheno-type assessment may have underestimated the number of fetuses, at 10 to 14 weeks, with chromosomal abnormalities that were normally aborted later, and overestimated the sensitivity test described by Pajkrt et al,^17^ leading to biased verification as highlighted by Begg & McNeil.^36^

In this study, an increase in fetal nuchal translucency, at 10 to 14 weeks’ gestation, showed a relationship with increased chromosomal alterations and was therefore useful in screening overall or individual chromosomal abnormalities. The accuracy was best for trisomy 21.

An important fact to be kept in mind is that the sample size for this study was calculated for overall chromosomal abnormalities and that an individualized analysis of the results for trisomy 21 would possibly have a statistical power inferior to that initially obtained.

The fact that this study analyzed a sample with a high risk for aneuploidy should be underscored, as it raised the accuracy of the screening tests. Consequently, this accuracy cannot be generalized for populations where the initial risk related to age and obstetric variables is smaller. It has been found that in neonates, polyploidy is extremely rare and that 30% of the fetuses with trisomy 21, 80% of those with trisomy 18, and nearly 99% of those with X monosomy are spontaneously aborted or evolve to death by 40 weeks of gestation.^35,38^ If these rates were applied to this study and the maximum loss rate for each aneuploidy was accepted, the NT screening would have identified 8 cases of fetal trisomy, 21 live births, 1 case of trisomy 18 and no cases of polyploidy or monosomy X.

In countries where abortion in legally allowed when chromosomal abnormalities are identified, research on screening tests is focused on trisomy 21 because of its low lethality.

In Brazil, the law does not provide for pregnancy interruption when chromosomal abnormalities are identified. However, a considerable number of these fetuses have had cardiac malformations or other defects in addition to aneuploidy, which can turn the gestational prognosis poor. The recognition of these fetuses with aneuploidy allows more specific examinations, such as fetal echocardiography and morphological ultrasound, that will assist in the risk classification of these fetuses, thereby allowing prenatal programs and delivery in tertiary services to be prepared to receive them. From a psychological point of view, screening and posterior diagnosis gives the couple the possibility of knowing the risks in the gestation and being ready for unfavorable situations, and it also helps to make them aware of the risks they face regarding future gestations.

In this study, the measurement of nuchal translucency demonstrated a high level of accuracy for a population that had a high prevalence of aneuploidy, and therefore it can be indicated in the same kind of circumstances.

## CONCLUSION

The measurement of nuchal translucency in a South American population showed a high degree of accuracy in screening overall chromosomal abnormalities and even higher accuracy for trisomy 21. The best cutoff point obtained for nuchal translucency was values ^3^ 2.5 mm.
